# Supplementary material for: Comparison of a Group-/Home-Based and a Weight-Machine-Based Exercise Training for Patients with Hip or Knee Osteoarthritis—A Secondary Analysis of Two Trial Interventions in a Real-World Context
Source: Int J Environ Res Public Health. 2022 Dec 19;19(24):17088. doi: 10.3390/ijerph192417088 (PMC9779110; doi:10.3390/ijerph192417088)
Supplement: Supplementary file 1 [file ijerph-19-17088-s001.zip › Supplement S1.pdf]

Supplement S1: In-and exclusion criteria for participation in the exercise interventions (regular font) and additional criteria for the accompanying study (*italic font*). Modified version of the original source: Krauss I, et al. BMJ Open Sport Exerc Med 2017; 3: e000291.

Inclusion criteria

- Hip or knee complaints (OA, state after joint-preserving or joint-replacing surgery and corresponding curative care, pre-arthritic conditions)
- Physical and mental ability to participate in the interventional program *and to answer self-administered questionnaires*
- *Self-reported lifetime prevalence of hip and/or knee OA diagnosed by a medical practitioner*
- *Insurance holder of the insurance company offering the exercise program for two or more years*

Exclusion criteria

- Significant established osteoporosis requiring treatment, previous spontaneous or low impact fracture
- Co-morbidities leading to major impairments in everyday life and representing contra-indications for physical activities
- Artificial joint replacement at the knee and/or hip joint within the last 6 months
- Artificial joint replacement at the knee and/or hip joint with instable anchoring
- Artificial joint replacement at the knee and/or hip joint with radiologic signs of implant loosening
- Current pain at rest or with activity due to artificial joint replacement at the knee and/or hip joint
- Luxation as an adverse event of artificial hip replacement
- Acute joint inflammation at the knee and/or hip joint
- Musculo-skeletal surgery at the lower extremity within the last 3 months
- Regular use of gait aids
- Self-reported acute illness
- Insufficient German language ability *for self-administered study questionnaires* and for the understanding of the verbal exercise instructions and written exercise descriptions and intervention materials.
- *Current employment in the health care insurance*
