# Supplementary material for: Comparison of a Group-/Home-Based and a Weight-Machine-Based Exercise Training for Patients with Hip or Knee Osteoarthritis—A Secondary Analysis of Two Trial Interventions in a Real-World Context
Source: Int J Environ Res Public Health. 2022 Dec 19;19(24):17088. doi: 10.3390/ijerph192417088 (PMC9779110; doi:10.3390/ijerph192417088)
Supplement: Supplementary file 1 [file ijerph-19-17088-s001.zip › Supplement S2.pdf]

Supplement S2: Completers vs. Dropouts

Variable	Overall (N=657)			By intervention group					
				HKT (N=521)			MbT (N=136)		
	**Completers** N=490 (74.6%)	**Dropouts** N=167 (25.4%)	**p**	**Completers** N=376 (83.8%)	**Dropouts** N=145 (27.8%)	**p**	**Completers** N=114 (72.2%)	**Dropouts** N=22 (16.2%)	**p**
**Sex n; %**									
Male	153 (31.2%)	35 (21.0%)	**.014**	97 (25.8%)	27 (18.6%)	.107	56 (49.1%)	8 (36.4%)	.387
Female	337 (68.8%)	132 (79.0%)		279 (74.2%)	118 (81.4%)		58 (50.9%)	14 (63.6%)	
**Age mean (sd)**	62.39 (10.08)	63.13 (10.8)	.436	63.41 (9.21)	63.95 (10.41)	.583	59.03 (11.98)	57.73 (11.95)	.644
**BMI mean (sd)**	28.06 (4.88)	27.48 (4.25)	.152	28.00 (4.78)	27.38 (4.24)	.159	28.28 (5.21)	28.14 (4.37)	.900
**Affected joint n; %**			**.049**			.067			.438
Knee	264 (54.6%)	77 (46.4%)		194 (52.3%)	66 (45.8%)		70 (62.5%)	11 (50.0%)	
Hip	110 (22.8%)	36 (21.7%)		91 (24.5%)	30 (20.8%)		19 (17.0%)	6 (27.3%)	
Both	109 (22.6%)	53 (31.9%)		86 (23.2%)	48 (33.3%)		23 (20.5%)	5 (22.7%)	
**Artificial joint n; %**			.281			.367			.696
Yes	65 (13.5%)	16 (9.8%)		52 (14.1%)	15 (10.6%)		13 (11.5%)	1 (4.8%)	
No	418 (86.5%)	147 (90.2%)		318 (85.9%)	127 (89.4%)		100 (88.5%)	20 (95.2%)	
Missings	7	4		6	3		1	1	
**Pain T0 mean (sd)**	3.00 (1.97)	3.57 (1.91)	**.001**	2.99 (2.02)	3.46 (1.83)	**.010**	3.03 (1.79)	4.25 (2.29)	**.025**
**Function T0 mean (sd)**	2.64 (1.91)	3.17 (1.88)	**.002**	2.62 (1.95)	3.09 (1.8)	**.001**	2.73 (1.75)	3.71 (2.32)	.071

HKT= Hip/knee Group training, MbT=Machine-based Training; SD=standard deviation

We found a statistically significant association between sex and study completion (p=.014). The data suggest that participants who dropped out were more frequently female (68.7% females in the completers vs. 79.0% females in the dropouts), however, this association did not reach statistical significance investigating dropouts within the two intervention groups separately. We also found an association between the site of OA and prematurely dropping out of the study, indicating higher drop-out rates for participants with OA at both, hip and knee joint (p=.049). This finding, however, could not be confirmed when we performed this analysis for each of the two interventions separately. Dropouts also exhibited significantly higher baseline WOMAC pain (mean=3.57 (SD=1.91) (dropouts) vs. mean=3.00 (SD=1.97) (completers); p=.001) and function scores (mean=3.17 (SD=1.88) (dropouts) vs. mean=2.64 (SD=1.91) (completers); p=.002).
