# Supplementary material for: Comparison of a Group-/Home-Based and a Weight-Machine-Based Exercise Training for Patients with Hip or Knee Osteoarthritis—A Secondary Analysis of Two Trial Interventions in a Real-World Context
Source: Int J Environ Res Public Health. 2022 Dec 19;19(24):17088. doi: 10.3390/ijerph192417088 (PMC9779110; doi:10.3390/ijerph192417088)
Supplement: Supplementary file 1 [file ijerph-19-17088-s001.zip › Supplement S3.pdf]

Supplement S3: Linear mixed models of WOMAC pain and function.

Variables	WOMAC pain (1p)			WOMAC function (1f)		
	Estimate	95% CI	p	Estimate	95% CI	p
Intercept	0.18	-0.63 – 1.00	0.664	-0.27	-1.02 – 0.49	0.490
T1 (vs T0)	0.12	-0.30 – 0.54	0.586	0.29	-0.09 – 0.67	0.136
Treatment GHT (vs MbT)	-0.20	-0.60 – 0.20	0.339	-0.01	-0.39 – 0.38	0.977
Baseline Severity [medium]	1.66	1.17 – 2.15	**<0.001**	1.67	1.22 – 2.12	**<0.001**
Baseline Severity [high]	4.06	3.57 – 4.55	**<0.001**	4.17	3.68 – 4.65	**<0.001**
Time [T1]* Treatment HKT	-0.01	-0.49 – 0.46	0.951	-0.08	-0.51 – 0.34	0.697
Time [T1]* Baseline Severity [medium]	-0.64	-1.20 – -0.07	**0.029**	-0.76	-1.25 – -0.27	**0.003**
Time [T1]* Baseline Severity [high]	-1.31	-1.90 – -0.72	**<0.001**	-1.28	-1.82 – -0.73	**<0.001**
Treatment [HKT]* Baseline Severity [medium]	0.04	-0.51 – 0.59	0.896	-0.07	-0.57 – 0.43	0.783
Treatment [HKT]* Baseline Severity [high]	0.22	-0.33 – 0.77	0.444	-0.19	-0.72 – 0.35	0.498
Time [T1]* Treatment [HKT]* Baseline Severity [medium]	0.05	-0.60 – 0.69	0.886	0.46	-0.11 – 1.02	0.116
Time [T1]* Treatment [HKT]* Baseline Severity [high]	-0.06	-0.73 – 0.60	0.856	0.47	-0.14 – 1.08	0.133
Age	0.01	0.00 – 0.02	**0.022**	0.01	0.00 – 0.02	**0.020**
Female (vs male)	0.04	-0.14 – 0.21	0.702	-0.02	-0.18 – 0.15	0.855
BMI	0.02	-0.00 – 0.03	0.062	0.02	0.01 – 0.04	**0.006**
OA site (vs knee)						
hip	-0.10	-0.31 – 0.10	0.330	-0.13	-0.32 – 0.06	0.178
both	0.01	-0.19 – 0.21	0.943	0.08	-0.10 – 0.27	0.389
Artificial joint	-0.00	-0.25 – 0.25	0.977	-0.14	-0.38 – 0.09	0.222

GHT = Group-/home-based training, MbT=Machine-based training.
